# Antiplatelet Agents Inhibit the Generation of Platelet-Derived Microparticles

**DOI:** 10.3389/fphar.2016.00314

**Published:** 2016-09-16

**Authors:** Alice Giacomazzi, Maurizio Degan, Stefano Calabria, Alessandra Meneguzzi, Pietro Minuz

**Keywords:** platelets, microparticles, thromboxane, ADP, collagen, fibrinogen receptor, collagen receptor

## Abstract

Platelet microparticles (PMPs) contribute to thrombogenesis but the effects of antiplatelet drugs on PMPs generation is undefined. The present study investigated the cellular events regulating PMPs shedding, testing *in vitro* platelet agonists and inhibitors. Platelet-rich plasma from healthy subjects was stimulated with arachidonic acid (AA), U46619, collagen type-I (10 and 1.5 μg/mL), epinephrine, ADP or TRAP-6 and pre-incubated with acetylsalicylic acid (ASA, 100 and 10 μmol/L), SQ-29,548, apyrase, PSB-0739, or eptifibatide. PMPs were detected by flow-cytometry using CD61 and annexin-V as fluorescent markers. Platelet agonists induced annexin V-positive PMPs shedding. The strongest response was to high concentration collagen. ADP-triggered PMPs shedding was dose-independent. ASA reduced PMPs induced by AA- (645, 347–2946 vs. 3061, 446–4901 PMPs/μL; median ad range, *n* = 9, *P* < 0.001), collagen 10 μg/mL (5317, 2027–15935 vs. 10252, 4187–46316 PMPs/μL; *n* = 13, *P* < 0.001), collagen 1.5 μg/mL (1078, 528–2820 vs. 1465, 582–5948 PMPs/μL; *n* = 21, *P* < 0.001) and TRAP-6 (2008, 1621–2495 vs. 2840, 2404–3031 PMPs/μL; *n* = 3, *P* < 0.01) but did not affect the response to epinephrine or ADP. The ADP scavenger apyrase reduced PMPs induced by U46619 (1256, 395–2908 vs. 3045, 1119–5494 PMPs/μL, *n* = 6, *P* < 0.05), collagen 1.5 μg/mL (1006, 780–1309 vs. 2422, 1839–3494 PMPs/μL, *n* = 3, *P* < 0.01) and TRAP-6 (904, 761–1224 vs. 2840, 2404–3031 PMPs/μL, *n* = 3, *P* < 0.01). The TP receptor antagonist SQ-29,548 and the P2Y_12_ receptor antagonist PSB-0739 markedly inhibited PMPs induced by low doses of collagen. Except for high-dose collagen, eptifibatide abolished agonist-induced PMPs release. Both TXA_2_ generation and ADP secretion are required as amplifiers of PMP shedding. The crucial role of the fibrinogen receptor and the collagen receptor in PMPs generation, independently of platelet aggregation, was identified.

## Introduction

Activated platelets vesiculate to produce platelet microparticles (PMPs), a heterogeneous population of small membrane-coated vesicles, ranging from 0.1 to 1.0 μm in diameter (Shai et al., 2012; Hsu et al., 2013). PMPs have been recognized as mediators of platelet and leukocyte adhesion, initiating and propagating coagulation and delivering to target cells membrane proteins along with cytosolic content including enzymes, mRNA, microRNA and, possibly, DNA (Merten et al., 1999; Ratajczak et al., 2006). Although the contribution of PMPs to hemostasis and thrombosis is still unclear, their procoagulant activity depends on the surface expression and assembly of proteins that are essential for coagulation and platelet activation, including various adhesion receptors, P-selectin and negatively charged phospholipids, mainly phosphatidylserine (Pasquet et al., 1996; Sinauridze et al., 2007; Burger et al., 2013).

PMPs represent approximately 70–90% of circulating microparticles in the blood of healthy individuals (Horstman, 1999; Diamant et al., 2004) and elevations of their levels have been detected in a number of disorders including cancer, atherosclerosis, sepsis, diabetes, acute coronary syndromes (Shantsila et al., 2010; Varon and Shai, 2015). Conversely, a deficiency in PMPs generation leads to a bleeding disorder with isolated prolonged bleeding time (Castaman et al., 1997). In the clinical setting, PMPs count may change in response to pharmacological treatment (Morel et al., 2006). Previous studies showed a reduction in PMPs generation in hyperlipidemic patients with type II diabetes after treatment with statins and eicosapentaenoic acid (Nomura et al., 2009) and also in patients with acute coronary syndromes treated with aspirin and P2Y_12_ receptor antagonists (Behan et al., 2005; Bulut et al., 2011). Furthermore, *in vitro* studies have shown an effective inhibition of shear- and agonist-induced PMP formation by inhibitory anti-GPIbα and anti-αIIbβ3 monoclonal antibodies (Gemmell et al., 1993; Pontiggia et al., 2006). Generation of PMPs, has been observed after chemical and physical platelet activation, either in association or not with platelet apoptosis (Zhang et al., 2013). The process of PMP shedding induced by platelet activation was demonstrated to be calcium-dependent (Heemskerk et al., 2002) and to be triggered by phospholipase C/inositol phosphate signaling (Bevers et al., 1989; Bird et al., 2004). Cytoskeleton rearrangement after the calpain cleavage of α-actinin, filamin, adducins, spectrin, talin is implicated (Fox et al., 1991). On the other hand, microvesiculation by apoptotic platelets results from a disruption of the balance between Bcl survival and Bak apoptotic signals (Mason et al., 2007; Zhang et al., 2007; Schoenwaelder et al., 2009), independently of platelet activation (Zhang et al., 2013).

PMPs formation can be induced *in vitro* by the activation of platelets with agonists (e.g., thrombin, collagen) (Takano et al., 2004) or compounds that directly target second messenger levels (e.g., calcium ionophores A23187, ionomycin) (Dachary-Prigent et al., 1995), phorbol esters and high shear stress (Holme et al., 1997), contact with artificial surfaces (Gemmell et al., 1995), complement (Sims et al., 1988) and low temperature (Bode and Knupp, 1994). Under experimental conditions an active metabolite of prasugrel was shown to strongly inhibit collagen and TRAP-induced PMPs formation (Judge et al., 2010).

As current knowledge about the signals underlying PMPs formation is still fragmentary, the present work further investigated the pathways involved in platelet microvesiculation also evaluating the modulation that antiplatelet agents may exert altering specific platelet functions. Particularly, the relative contribution of platelet amplification signals, such as endogenous thromboxane A_2_ (TXA_2_) and secretion of ADP, and the role of integrin αIIbβ3 and the GPVI-α2/β1 complex in agonist-induced PMPs shedding were evaluated *in vitro* along with the effects of platelet inhibitors. To this aim we developed a protocol for testing *in vitro* agonist-induced PMPs generation using a flow cytometry (FCM)-based analysis (Robert et al., 2009).

## Materials and Methods

### Ethical Statement

The use of platelet rich plasma from healthy donors for *in vitro* studies was approved by the local Ethical Committee (Comitato Etico per la Ricerca Clinica delle Province di Verona e Rovigo).

### Blood Samples

Venous blood was obtained in the morning (between 9 and 11 a.m.) from healthy and fasting volunteers who gave their informed consent and had not taken any drugs affecting platelet function in the previous 2 weeks. A clean puncture of an antecubital vein was performed with a 20-gauge needle (Safety^®^-Multifly-Set, Sarstedt, Nümbrecht, Germany) following the application of a light tourniquet, while blood collection was performed without applying venostasis. After discarding of the first 2–3 ml of blood, S-Monovette^®^ tubes (Sarstedt) containing 100 μmol/L PPACK (Enzo Life Sciences Inc., Farmingdale, NY, USA) were used as collection tubes and anticoagulant was immediately mixed with blood by gentle inversion. PPACK was used as anticoagulant in order to maintain physiological calcium concentration in plasma. Transportation of blood tubes to the laboratory was careful to avoid unnecessary agitation; for this purpose, a box maintaining the tubes in a steady vertical position was used. Samples were kept at room temperature (20–24°C) and the delay before the first centrifugation was less than 1 h.

### Preparation of Platelet-Rich Plasma (PRP), Platelet Activation, and Microparticle Formation

Platelet-rich-plasma (PRP) was prepared after venipuncture by centrifugation of blood at 180 × *g* × 15 min at room temperature and transferred to polypropylene tubes, leaving 1 cm of PRP above the buffy layer and taking care not to disturb it. To induce PMPs shedding, platelets were activated in the absence or presence of antiplatelet agents. Particularly, aliquots (500 μL) of PRP were stimulated by incubation with various agonists for 30 min at room temperature under low shear stress conditions (approximately 1 dyne/cm^2^) using a GyroMini^TM^ Nutating Mixer (Labnet Int. Edison, NJ, USA). These experimental conditions were chosen to limit the formation of platelet aggregates, in order to obtain more specific information concerning defined processes, e.g., P-selectin or CD40L expression (Furman et al., 2004; Hu et al., 2004). The following platelet agonists were used: 1.25 mmol/L arachidonic acid (AA; Cayman Chemical, Ann Arbor, MI, USA), 2 μmol/L U46619 (Calbiochem, Merck-Millipore, Billerica, MA, USA), 1.5 μg/mL and 10 μg/mL collagen type I (Mascia Brunelli S.p.a, Milano, Italy), a combination of low doses of collagen type I and 10 μmol/L epinephrine (Calbiochem), 10 μmol/L epinephrine, 0.5 1 2–5 μmol/L ADP (Mascia Brunelli S.p.A) and 20 μmol/L TRAP-6 (Tocris Bioscience, R&D Systems, Minneapolis, MN, USA). Saline solution 0.9% NaCl (Fresenius Kabi Italia S.r.l, Verona, Italy) was used instead of agonists for the resting conditions. In order to evaluate the effects of platelet inhibitors on *in vitro* PMPs generation, freshly isolated PRP was pre-incubated with a highly selective TP receptor antagonist 20 μmol/L SQ-29,548 (Cayman Chemical), a scavenger of ADP 10 U/mL apyrase (Sigma-Aldrich, St. Louis, MO, USA), a P2Y_12_ receptor antagonist 500 nmol/L PSB-0739 (Tocris Bioscience), and the αIIbβ3 antagonist 10 μg/mL eptifibatide (Selleckchem, Munich, Germany), a synthetic RGD mimetic, which has previously been used to prevent the activation of the fibrinogen receptor (Furman et al., 2004; Minuz et al., 2006). Incubation was for 30 min at room temperature and PRP was subsequently stimulated as described above. Acetylsalicylic acid (ASA 10 and 100 μmol/L) was added immediately after blood sampling to allow the irreversible acetylation of platelet cyclooxygenase type 1.

### PMPs Preparation and Labeling

After activation, platelet-free plasma (PFP) samples were prepared by centrifugation of PRP at 13000 × *g* for 5 min at room temperature, avoiding application of the centrifuge brake. A platelet pellet was evident at the bottom of the plastic tubes while PMPs remained in the supernatant.

For PMPs labeling, 20 μL of PFP was carefully removed after centrifugation and added to 80 μL of calcium-rich binding buffer containing 5 μL annexin V-fluorescein isothiocyanate (FITC; from Annexin V-FITC Apoptosis Detection Kit, eBioscience, San Diego, CA, USA) and 5 μL of anti-human CD61 or, alternatively, anti-human CD41-phycoerythrin (PE; BioLegend, San Diego, CA, USA). Anti-CD61 (or CD41) was used to confirm platelet origin and gate out any artifact. A PE Mouse IgG_1_ (IgG_1_ K, clone MOPC-21, BioLegend) antibody was used as isotype control. After 30 min of incubation in the dark at room temperature, samples were diluted in 1 mL Hepes buffer (10 mmol/L HEPES, 6 mmol/L glucose, 145 mmol/L NaCl, 5 mmol/L KCl, 0.5 mmol/L NaH_2_PO_4_, pH 7.4) containing 3 mmol/L CaCl_2_. Finally 25 μL of counting beads with an established concentration close to 1000 beads/μL (Flow-Count^TM^ Fluorospheres, Beckman Coulter, Pasadena, CA, USA) was added to each sample in order to express PMPs count as absolute numbers per microliter of PFP. A concentration-matched isotype antibody (IgG_1_-PE, BioLegend) or annexin V-FITC with phosphate-buffered saline (PBS, Sigma-Aldrich) without calcium were used as controls for CD61 (or CD41)-PE and annexin V-FITC, respectively. PMPs were finally analyzed by FCM as CD61 (or CD41)-PE/annexin V-FITC-positive events in the MP region.

### Flow Cytometric Analysis

Analyses of labeled samples were performed on a Cytomics FC500 flow-cytometer (Beckman Coulter) as previously described (Robert et al., 2009). Briefly, after standardization of the protocol with a blend of monodisperse fluorescent beads of three diameters (0.5, 0.9 and 3 μm, Megamix, Stago, Biocytex, Marseille, France), optimal instrument settings and the MP region were defined. Megamix beads were run before starting each analysis in order to control and, eventually, to adjust FCM-settings. Forward (FS) and side (SS) scatter parameters were plotted on logarithmic scales to best cover a wide size range. PMPs were gated in the MP window and defined as single CD61 (or CD41)+ events or dual-positive phosphatidylserine (PS)^+^/CD61 (or CD41)^+^ events, as seen in dual-color fluorescence plots after staining with annexin V-FITC and CD61 (or CD41)-PE. Single staining controls were used to check fluorescence compensation settings and to set up positive regions. Each tube was run for 1 min at medium flow-rate, with a maximum delay of 30 min after the end of staining.

To limit background noise from dust and crystals, flow cytometric analyses were performed using a 0.22 μm-filtered sheath fluid (Isoflow^TM^, Beckman Coulter). CXP ACQUISITION and CXP ANALYSIS software packages (Beckman Coulter) were used for data acquisition and analysis, respectively.

### Statistical Analysis

For statistical analysis, all data were analyzed with GraphPad Prism software v.5.03 (GraphPad Software, San Diego, CA, USA), expressed as PMPs absolute count per microliter of PFP. The Mann–Whitney test was used in all the experiments to evaluate differences between two groups and the Kruskall–Wallis test was applied for multiple comparisons, with Dunn’s test as *post hoc* analysis. Data represent *n* repeats of each experiment performed in duplicate and are expressed as median and range in figures (with interquartiles) and text, mean and standard deviation in tables. *P* < 0.05 was assumed as statistically significant.

## Results

### Definition of Standardized FCM Settings for PMP Analysis

Before starting all the analysis of PMPs, FCM settings and MP analysis region were established and standardized for PMPs counts according to a FCM-based protocol (Robert et al., 2009). Validation of the data was performed before each single analysis by using Megamix beads, allowing us to discriminate between beads <1.0 μm on the basis of size. A clear discrimination between fluorescent beads of 0.5, 0.9, and 3.0 μm in size was evident. Therefore, this calibrated-bead strategy allowed us to focus our analysis on PMPs in a reproducible size range (0.5–0.9 μm).

### *In vitro* Agonist-Induced PMP Generation

**FIGURE 1:**
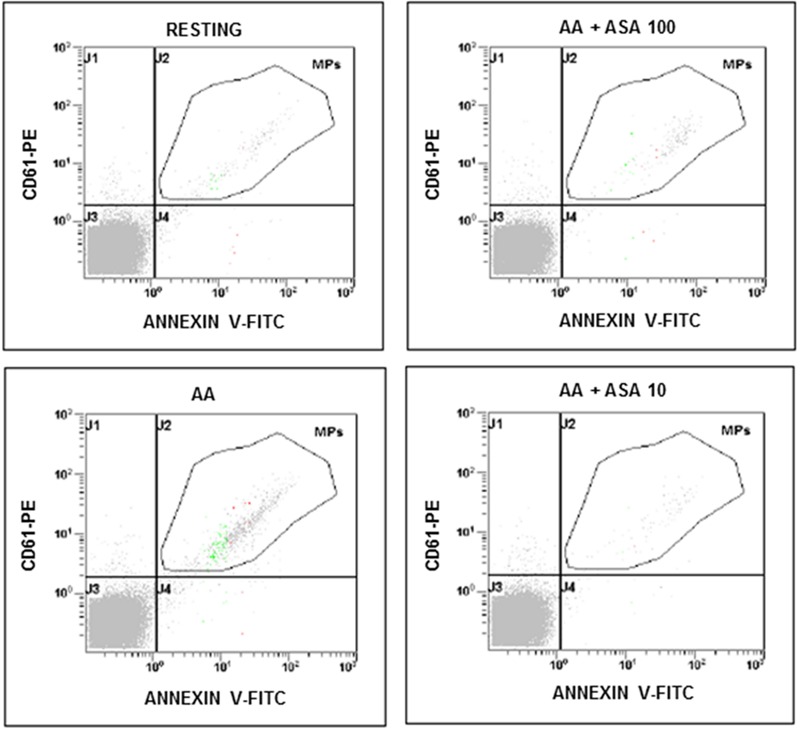
**Flow cytometric analysis of platelet-derived microparticles shed after platelet stimulation with arachidonic acid (AA; 1.25 mmol/L) and in the presence of different concentrations of ASA (100 and 10 μmol/L).** Dot plots show the dual fluorescence analysis of representative PFP stained with Annexin V-fluorescein isothiocyanate (FITC) and anti-CD61-phycoerythrin (PE). The total number of CD61+ MPs was calculated as the sum of CD61+/Annexin and CD61+/Annexin + MPs. Absolute counts of PMPs were determined by using Flow-Count^TM^ Fluorospheres (Beckman Coulter) and expressed per microliter of PFP. Experiments were performed in duplicate.

**FIGURE 2:**
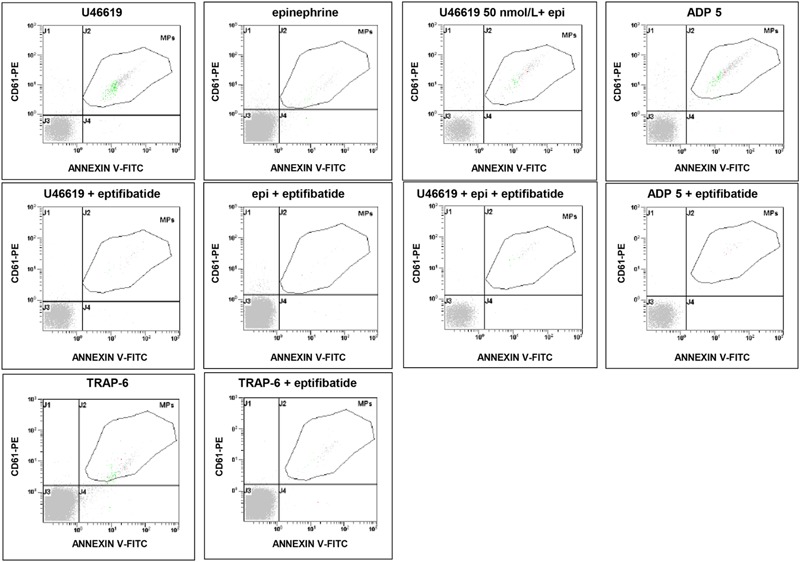
**Flow cytometric analysis of platelet-derived microparticles shed after pre-incubation of platelets with eptifibatide (10 μg/mL) and stimulation with different platelet agonists.** Dot plots show the dual fluorescence analysis of representative PFP stained with Annexin V-FITC and anti-CD61-phycoerythrin (PE). The total number of CD61+ events was calculated as the sum of CD61+/Annexin and CD61+/Annexin + events. Absolute counts of PMPs were determined by using Flow-Count^TM^ Fluorospheres and expressed per microliter of PFP. Experiments were performed in duplicate.

**FIGURE 3:**
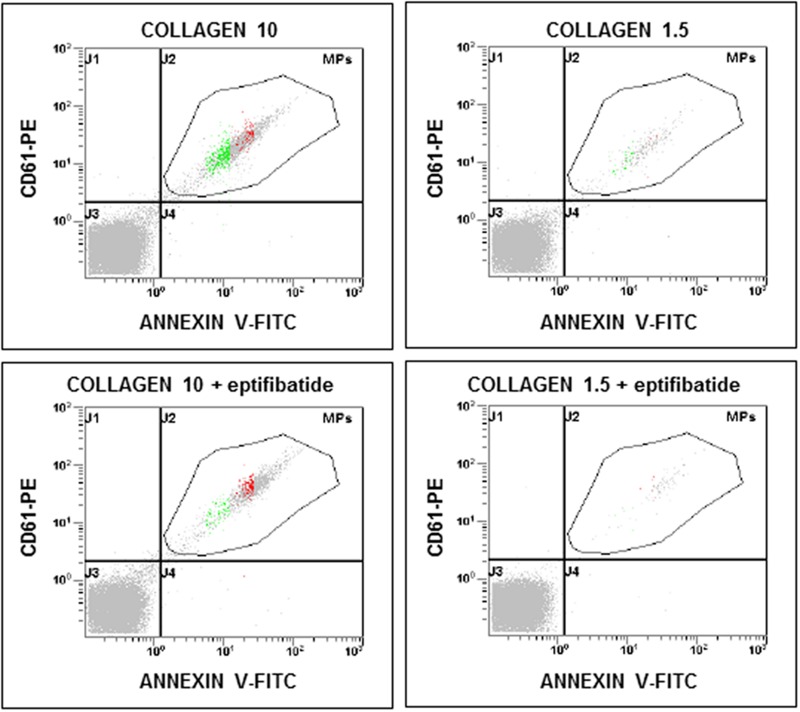
**Flow cytometric analysis of platelet-derived microparticles shed after pre-incubation of platelets with eptifibatide (10 μg/mL) and stimulation with different concentrations of collagen type I.** Dot plots show the dual fluorescence analysis of representative PFP stained with Annexin V-FITC and anti-CD61-phycoerythrin (PE). The total number of CD61+ events was calculated as the sum of CD61+/Annexin and CD61+/Annexin + events. Absolute counts of PMPs were determined by using Flow-Count^TM^ Fluorospheres and expressed per microliter of PFP. Experiments were performed in duplicate.

**FIGURE 4:**
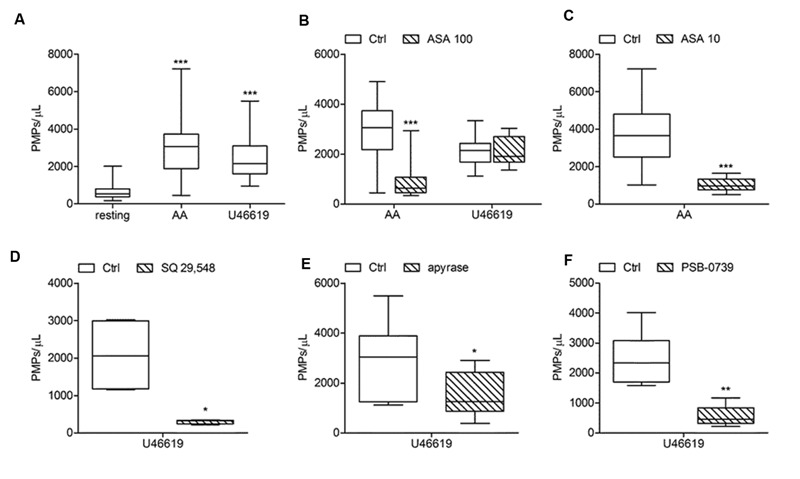
**Effects of antiplatelet agents on *in vitro* platelet microparticle generation induced by AA and U46619. (A)** *In vitro* PMPs generation induced by AA (1.25 mmol/L) and U46619 (2 μmol/L) in freshly isolated PRP under low shear stress conditions. **(B,C)** Effects of acetylsalicylic acid (ASA) at high (100 μmol/L) and low (10 μmol/L) concentration on platelet microvesiculation triggered by AA and U46619. **(D,E,F)** Effects of SQ-29,548 (20 μmol/L), apyrase (10 U/mL) and PSB-0739 (500 nmol/L) on *in vitro* PMP shedding induced by U46619. (*n* = 3–20, *^∗^P* < 0.05; ^∗∗^*P* < 0.01; ^∗∗∗^*P* < 0.001).

**FIGURE 5:**
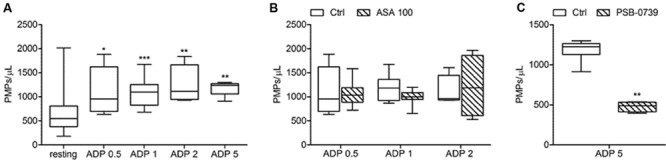
***In vitro* ADP-induced platelet microvesiculation and effects of platelet inhibitors. (A)** PMPs generation induced by different concentrations of ADP (0.5, 1, 2, 5 μmol/L) in freshly isolated PRP under low shear stress conditions; **(B)** Effects of high concentration of ASA (100 μmol/L) on ADP-triggered PMP shedding; **(C)** Effect of PSB-0739 (500 nmol/L) on PMP generation induced by platelet stimulation with ADP. (*n* = 4). *^∗^P* < 0.05; ^∗∗^*P* < 0.01; ^∗∗∗^*P* < 0.001.

**FIGURE 6:**
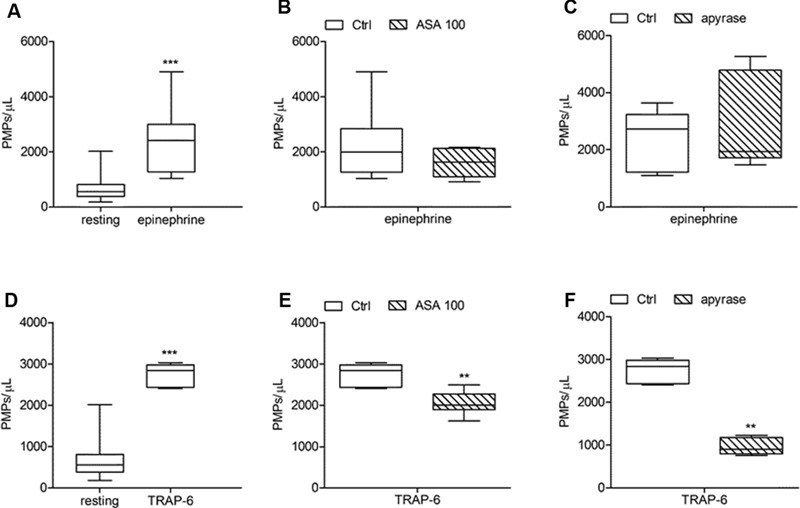
**Effects of ASA and apyrase on *in vitro* PMPs shedding induced by platelet stimulation with epinephrine and TRAP-6. (A,D)** *In vitro* PMPs generation induced by epinephrine (10 μmol/L) and TRAP-6 (20 μmol/L) in freshly isolated PRPs under low shear stress conditions; **(B,E)** Effects of high concentration of ASA (100 μmol/L) on epinephrine- and TRAP-6-triggered PMP shedding; **(C,F)** Effects of apyrase (10 U/mL) on platelet microvesiculation induced by epinephrine and TRAP-6. (*n* = 3–6). ^∗∗^*P* < 0.01; ^∗∗∗^*P* < 0.001.

**FIGURE 7:**
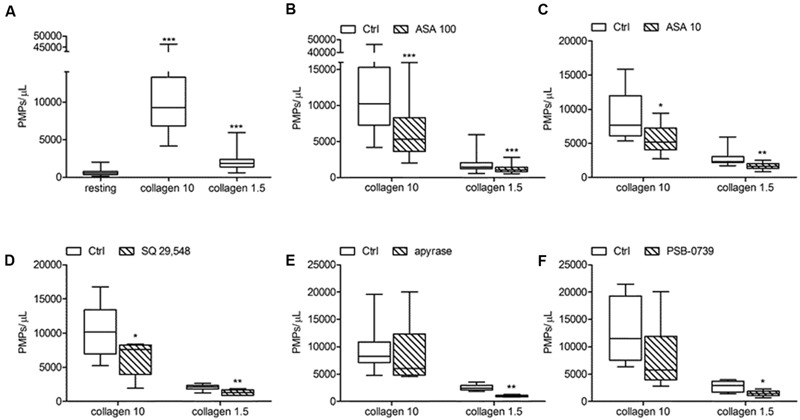
***In vitro* collagen-induced platelet microparticle generation and effects of antiplatelet agents. (A)** PMP generation induced by collagen type I at high (10 μg/mL) and low (1.5 μg/mL) concentration in freshly isolated PRP under low shear stress conditions; **(B,C)** Effects of ASA at high (100 μmol/L) and low (10 μmol/L) concentration on platelet microvesiculation triggered by collagen type I (10 and 1.5 μg/mL). **(D,E,F)** Effects of SQ 29,548 (20 μmol/L), apyrase (10 U/mL) and PSB-0739 (500 nmol/L) on *in vitro* PMPs shedding induced by collagen type I (10 and 1.5 μg/mL). *^∗^P* < 0.05; ^∗∗^*P* < 0.01; ^∗∗∗^*P* < 0.001.

In our experiments PMPs were detected in the particulate fraction shed from *in vitro* activated platelets and identified by FCM as small-size scatter events staining positive for integrin β3 (CD61), or integrin αIIb (CD41). Preliminary observations demonstrated that in the presence of platelet agonists the application of low shear stress was necessary for PMPs shedding (**Table 1**). Stimulation of freshly isolated PRP under low shear stress conditions (approximately 1 dyne/cm^2^) with any of the platelet agonists increased generation of PMPs compared to the resting conditions, where a limited number of microvesicles were shed (**Figures 1–3**; **Table 2**). We observed that most of the CD61 (or CD41) positive events stained with fluorescent-labeled annexin V, marker of exposed phosphatidylserine (PS) on platelet-derived MP surface (**Figures 1–3**; **Table 2**). We observed that under resting condition 65% of PMPs were annexin V-positive; when platelets were incubated with agonists, such as 1.25 mmol/L AA and the stable TXA_2_ analog U46619, 2 μmol/L, a significant increase in PMPs shedding was observed, with an increase to 85% of annexin V-positive PMPs (**Table 2**; **Figure 4A**). Compared to resting conditions, in the presence of different concentrations of ADP (0.5–1.0–2.0–5.0 μmol/L), platelets shed a larger amounts of microparticles in a dose-independent manner (**Table 2**; **Figure 5A**). Microvesicle formation was also significantly enhanced by epinephrine, either alone (10 μmol/L) (**Table 2**; **Figure 6A**) or associated with collagen 1.5 μg/mL (2117, 1563–2583 PMPs/μL vs. 552, 180–2016 PMPs/μL, *P* < 0.05) and by TRAP-6 (**Table 2**; **Figure 6D**). Collagen, at the highest tested doses, proved to be the strongest trigger for the release of PMPs, of which 95% were annexin V-positive (**Table 2**; **Figure 7A**).

**Table 1:** Platelet MicroParticles (PMPs) generation under static and under flow conditions.

Agonist	Static conditions (PMPs/μL, Median and Range)	Low Shear Stress (PMPs/μL, Median and Range)	Static vs. Low Shear
None (resting PRP)	134, 113–224 (*n* = 4)	308, 170–709 (*n* = 4)	*p* < 0.01
Arachidonic acid 1,25 mmol/L	455, 208–953 (*n* = 4)	1388, 873–3200 (*n* = 4)	*p* < 0.001
Collagen type I (10 μg/mL)	619, 320–1643 (*n* = 4)	5936, 3191–12987 (*n* = 4)	*p* < 0.001

*PMPs generation was evaluated after incubation of freshly isolated PRP with saline (resting PRP), arachidonic acid (1.25 mmol/L) and collagen-type I (10 μg/mL) under static or low shear stress conditions for 30 min at RT. PMPs were detected by flow cytometry (FCM) and identified as double staining positive events for annexin V-fluorescein isothiocyanate (FITC) and anti-CD41-phycoerythrin (PE) mAb. Data are expressed as PMPs absolute counts per microliter of PFP and reported as median and range of *n* duplicate experiments.*

**Table 2:** Effects of platelet agonists on PMPs generation.

Agonist	PMPs/μL (CD61+)	PMPs/μL (CD61+/annexinV+)
Resting platelets	948 ± 667 (*n* = 53)	652 ± 376 (*n* = 53)
Arachidonic acid 1.25 mmol/L	3479 ± 1730 (*n* = 15)	2980 ± 1473 (*n* = 15)
U46619 2 μmol/L	2773 ± 1240 (*n* = 20)	2431 ± 1098 (*n* = 20)
Collagen type I 1.5 μg/mL	2343 ± 1127 (*n* = 41)	1973 ± 897 (*n* = 41)
Collagen type I 10 μg/mL	11630 ± 7557 (*n* = 33)	11080 ± 7410 (*n* = 33)
Epinephrine 10 μmol/L	3112 ± 1593 (*n* = 8)	2296 ± 1090 (*n* = 8)
ADP 0.5 μmol/L	1233 ± 626 (*n* = 3)	1115 ± 494 (*n* = 3)
ADP 1 μmol/L	1217 ± 369 (*n* = 5)	1081 ± 315 (*n* = 5)
ADP 2 μmol/L	1627 ± 701 (*n* = 3)	1258 ± 385 (*n* = 3)
ADP 5 μmol/L	1360 ± 159 (*n* = 3)	1182 ± 155 (*n* = 3)
TRAP-6 20 μmol/L	3162 ± 490 (*n* = 3)	2753 ± 272 (*n* = 3)

*PMPs were detected by FCM and identified as single staining positive events using anti-CD41-phycoerythrin (PE) mAb double staining positive events for annexin or double positive using V-FITC and anti-CD41-phycoerythrin (PE) mAb. Data are expressed as PMP absolute counts per microliter of PFP and reported as median and range of *n* duplicate experiments.*

### Effects of Acetylsalicylic Acid and SQ-29,548 on Agonist-Induced Platelet Microvesiculation

The effects of ASA (100 and 10 μmol/L) on PMPs generation induced by agonists exploring the TXA_2_ – TP receptor pathway was investigated in PPACK-anticoagulated PRP (**Figures 4A,D**). More in details, we found that platelet microvesiculation induced by AA (1.25 mmol/L) was significantly reduced when 100 μmol/L ASA was added at the time of blood sampling (**Figure 4B**). Similar were the effects of a lower concentrations of aspirin (10 μmol/L) on PMPs generation induced by AA (**Figure 4C**). As expected, U46619-induced platelet microvesiculation was unchanged in the presence of ASA (**Figure 4B**). The role of TXA_2_ in collagen-induced platelet microvesiculation was further confirmed by using SQ-29,548 a highly selective TP receptor antagonist, which almost completely abolished the effect of U46619 (**Figure 4D**).

Acetylsalicylic acid had no effects on PMPs shedding from platelets stimulated either with all the tested concentrations of ADP (0.5–1.0–2.0 μmol/L) (**Figures 5A,B**), epinephrine alone (**Figures 6A,B**), or the combination of 1.5 μg/mL collagen and 10 μmol/L epinephrine (1169, 831–2457 PMPs/μL vs. 2117, 1563–2583 PMPs/μL, *P* = n.s.). However, ASA significantly reduced PMPs generation induced by TRAP-6 (**Figure 6**) and collagen (1.5 and 10 μg/mL) (**Figures 7A–C**). We also found a significant decrease in PMP formation induced by collagen (10 μg/mL and 1.5 μg/mL) after pretreatment of PRP with SQ-29,548, as shown in **Figure 7D**.

### Effects of Apyrase and PSB-0739 on PMPs Generation

The relative contribution of endogenous ADP on agonist-induced PMPs generation was evaluated by using apyrase, a scavenger of ADP. In the presence of 10 U/mL apyrase decreased PMPs shedding after stimulation with U46619 (**Figures 4A,E**), TRAP-6 (**Figure 6F**), or low concentration collagen (1.5 μg/mL) was observed (**Figure 7E**), while apyrase did not significantly affect platelet responses to epinephrine (**Figure 6C**) and 10 μg/mL collagen (**Figure 7E**). Moreover, apyrase reduced the number of PMPs in PFP obtained from unstimulated PRP (357, 164–1238 PMPs/μL vs. 745, 213–2016 PMPs/μL, *P* < 0.05).

The use of PSB-0739, a highly potent P2Y_12_ receptor antagonist, further confirmed these results, also indicating the role of this receptor in the pathway leading to microparticle generation. As expected, the effect of ADP (5 μmol/L) was completely abolished (**Figure 5C**). The release of MPs from platelets activated with U46619 (**Figure 4F**) and low concentration collagen (**Figure 7F**) was markedly reduced after incubation of PRP with PSB-0739, while there were no significant effects on PMP generation induced by 10 μg/mL collagen (**Figure 7F**). Moreover, similarly to what observed in the presence of apyrase, PSB-0739 reduced PMPs count in resting conditions (data not shown).

Finally, the combined use of ASA and PSB-0739 (1222, 539–2602 PMPs/μL) on PMP shedding induced by low concentration collagen (1.5 μg/mL) did not shown any additional effect compared to PSB-0739 (1437, 709–2252 PMPs/μL) or ASA alone (1587, 1086–2284 PMPs/μL, *n* = 4, *P* = n.s.).

### Effects of Eptifibatide on Agonist-Induced Generation of PMPs

**FIGURE 8:**
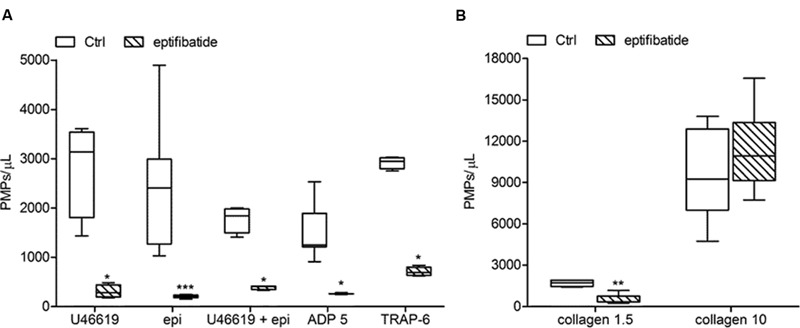
**Effects of eptifibatide on *in vitro* agonist-induced platelet microvesiculation. (A,B)** Effects of eptifibatide (10 μg/mL) on PMPs generation induced by different soluble agonists (U46619, 2 μmol/L; epinephrine, epi, 10 μmol/L; U46619, 50 nmol/L+epinephrine, 10 μmol/L; ADP, 5 μmol/L, TRAP-6, 20μmol/L) and collagen type I (1.5 and 10 μg/mL) in freshly isolated PRP under low shear stress conditions. (*n* = 4). *^∗^P* < 0.05; ^∗∗^*P* < 0.01; ^∗∗∗^*P* < 0.001.

In order to evaluate the potential implication of integrin αIIbβ3 on PMPs formation, freshly isolated PRP was pre-incubated with eptifibatide (10 μg/mL) and then stimulated with platelet agonists. As shown in **Figure 8A**, in the presence of this GPIIb/IIIa antagonist, a strong inhibition of platelet microvesiculation was found after stimulation of PRP with soluble agonists, such as ADP (5 μmol/L), U46619, TRAP-6 and epinephrine (10 μmol/L), either alone or in combination with low concentration U46619 (50 nmol/L). Similarly, eptifibatide reduced the release of PMPs from platelets stimulated with collagen 1.5 μg/mL, but did not affect the response to high concentration collagen (10 μg/mL) (**Figure 8B**). A significant reduction of PMP generation was also observed in resting conditions after incubation with eptifibatide (180, 85–403 PMPs/μL vs. 743, 369–2016 PMPs/μL, *P* < 0.0001). Finally, none of the platelet inhibitors altered the ratio of annexin V-positive to annexin V-negative PMPs (data not shown).

## Discussion

In the present work, we used an *in vitro* FCM-based protocol (Robert et al., 2009) to evaluate the effects of antiplatelet agents on agonist-induced PMPs shedding and their procoagulant properties. By using specific agents affecting platelet function we obtained clues concerning the signaling pathways involved in platelet microvesiculation, demonstrating the crucial role of mediators of the amplification process and integrin implication. The results from the present study confirm that suspended platelets shed microparticles when activated *in vitro* with soluble agonists (Fox et al., 1991; Barry et al., 1997; Perez-Pujol et al., 2007). Therefore, procoagulant activity is induced, as assessed by evaluating the binding of annexin V as an index of phosphatidylserine (PS) expression that increased after exposure to platelet agonists, considering percentage and total number of annexin V-positive PMPs (Pasquet et al., 1996; Sinauridze et al., 2007; Connor et al., 2010). We observed that platelets generate PMPs, both when activated by strong agonists, as previously show with thrombin, collagen and calcium ionophores (Fox et al., 1991; Barry et al., 1997; Perez-Pujol et al., 2007), and by weak agonists, such as ADP and epinephrine (Judge et al., 2010; Zhang et al., 2013). In addition, we found that shear stress is required for platelet microvesiculation. The observed inability of both resting and agonist-stimulated platelets to shed PMPs when shear stress was not applied, is consistent with recent evidence indicating that strong agonists, such as thrombin and collagen, although unable to induce significant platelet procoagulant activity under static conditions, promote high levels of PS exposure and microvesiculation under physiological levels of shear stress (Delaney et al., 2014). According to previous studies, high shear stress is required to induce von Willebrand factor (vWF)-dependent glycoprotein Ibα-mediated platelet procoagulant activity (Reininger et al., 2006) and exposure of platelets to extremely high shear stress is sufficient to induce platelet microvesiculation, independently of any stimulation with soluble agonists (Holme et al., 1997; Reininger et al., 2006).

Antiplatelet agents modulate PMPs generation acting on intracellular signaling pathways involved in this process. Pretreatment of platelets with ASA or SQ-29,548 results in a significant reduction of microvesiculation induced by the tested agonists, except for ADP and epinephrine. ASA, particularly at the lower tested concentration (10 μmol/L), specifically inhibits platelet function by acetylating cyclooxygenase-1 (COX-1) causing the irreversible inhibition of thromboxane generation, while SQ-29,548 is a highly selective TP receptor antagonist. The inhibitory effects obtained with these agents clearly suggest a role of endogenous TXA_2_ on agonist-triggered PMP shedding. The contribution of secreted ADP was assessed by using apyrase, an enzyme with ADP-scavenging activity, and PSB-0739, a highly potent P2Y_12_ receptor antagonist (Hoffmann et al., 2009). Under these conditions, a significant decrease in PMP shedding from platelets stimulated with U46619 and low collagen was observed, not with high concentration collagen. Interestingly, pre-treatment with apyrase and PSB-0739 reduced also the number of PMPs in PFP obtained from unstimulated PRP, indicating that under these experimental conditions released ADP accounts for a limited platelet microvesiculation. Since the effects of apyrase and those of PSB-0739 were superimposable, we can affirm that P2Y_12_ engagement is required for PMPs generation in response to agonists. Cooperative signals of ADP and TXA_2_ contribute to platelet MP release and this is similar to the strict connection of activatory pathways that occurs in platelet amplification process and aggregation, where released ADP cooperates to induce TXA_2_ biosynthesis and ADP-dependent signals are required for TXA_2_-dependent platelet activation (Minuz et al., 2006). Therefore, in addition to the ability of exogenous TXA_2_ and ADP to directly promote *in vitro* PMPs shedding, the present findings reveal their autacoid function and the contribution of platelet secretion to PMPs generation. Other signaling pathways are implicated in microvesiculation, as indicated by data obtained using epinephrine as platelet agonist showing that the generation of procoagulant PMPs does not require ADP nor TXA_2_. Although data concerning released ADP, as assessed by testing the effects of apyrase, seem to implicate a necessary role for platelet secretion, a dissociation between granule secretion and PMPs generation has been recently demonstrated (Delaney et al., 2014). Concerning the signaling pathways implicated, the small GTPase Rac1 was found to play a central role in mediating the procoagulant response and PMPs release induced by low-dose of soluble agonists (Delaney et al., 2014).

Previous studies indicate that a larger number of PMPs is released from suspended platelets than from platelets adhering to immobilized substrates (Zhang et al., 2013). Under static conditions, when platelet spreading occurs, microvesiculation is associated to a change in platelet morphology, independently from implication of the fibrinogen receptor suggesting that integrin activation is not required (Zhang et al., 2013). Here we show that activation of the fibrinogen receptor, is necessary for the generation of procoagulant PMPs in suspended platelets stimulated with agonists acting on platelets G-coupled receptors. In fact, this phenomenon is completely prevented by the ligation of the active fibrinogen receptor by eptifibatide, a specific small-molecule inhibitor of the αIIbβ3 complex. Previous studies have shown that eptifibatide at the concentrations used in the present study completely prevents the activation of the αIIbβ3 complex, as assessed using the monoclonal antibody PAC-1, which specifically binds the active fibrinogen receptor, thus preventing downstream activatory signaling and platelet aggregation (Furman et al., 2004; Minuz et al., 2006). However, platelet aggregation is not required for PMPs release. This is supported by the evidence that microparticle generation occurs also in the presence of eptifibatide when high concentration collagen is used as a stimulus. A dissociation between platelet aggregation and PMPs generation is also consistent with previous evidence that inhibition of RhoA prevents thrombin–induced platelet aggregation, but neither phosphatidylserine exposure nor microvesiculation are altered (Delaney et al., 2014).

Therefore, our results indicate that both integrin αIIbβ3 and glycoprotein Ibα are implicated in PMPs release and show that ligation of the GPVI-α2/β1 complex under shear stress generates PMPs (Gemmell et al., 1993; Boilard et al., 2010; Hsu et al., 2013). Our data identify a specific mechanism for collagen-induced PMPs generation in suspended platelets. At low doses, collagen requires both TXA_2_ and ADP to generate PMPs, but at high doses proves to be *per se* the strongest of the tested agonists also when TXA_2_-and ADP-dependent pathways are inhibited. Therefore, ligation of the GPVI-α2/β1 complex induces PMPs generation by activating signaling pathways that are substitute for signals downstream αIIbβ3 activation.

New in the present study are the systematic investigation into the effects of different antiplatelet agents on microparticle generation and the demonstration that inhibition of individual pathways blunts the generation of procoagulant microprticles, giving clues for further basic and clinical investigation. Showing that activation of the AA pathway and the release of ADP from delta granules amplify the release of microvesicles from platelets, we speculate that both aspirin and P2Y_12_ inhibitors may blunt also *in vivo* the prothrombotic potential of activated platelets. Moreover, since PMPs generation is strongly dependent on integrin engagement, both inhibitors of the fibrinogen and the collagen receptors may also prove effective *in vivo*. Modulation of PMPs generation may explain part of the antithrombotic activity that antiplatelet agents exert in different clinical settings, including those unrelated to atherosclerosis, Moreover, since PMPs may have a pathogenetic role in different experimental and clinical conditions, including inflammatory diseases (Boilard et al., 2010) and cancer (Varon and Shai, 2015), beneficial effects of antiplatelet intervention is expected, beyond inhibition of thrombus formation.

## Author Contributions

PM designed the study, analyzed data and wrote the manuscript; AG was the main investigator taking part to study design, performed the experiments, and wrote the manuscript; MD designed the protocol for the flow-cytometry analysis of platelet microparticles and performed the experiments and data analysis. SC and AM took part in study design, experiments, data analysis and the preparation of the manuscript.

## Conflict of Interest Statement

The authors declare that the research was conducted in the absence of any commercial or financial relationships that could be construed as a potential conflict of interest.

The reviewer MD and handling Editor declared their shared affiliation, and the handling Editor states that the process nevertheless met the standards of a fair and objective review.
